# A Brief Overview of the Antitumoral Actions of Leelamine

**DOI:** 10.3390/biomedicines7030053

**Published:** 2019-07-19

**Authors:** Myriam Merarchi, Young Yun Jung, Lu Fan, Gautam Sethi, Kwang Seok Ahn

**Affiliations:** 1 Faculty of Pharmacy, University of Paris Descartes, 75006 Paris, France; 2 Department of Pharmacology, Yong Loo Lin School of Medicine, National University of Singapore, Singapore 117600, Singapore; 3 College of Korean Medicine, Kyung Hee University, 24 Kyungheedae-ro, Dongdaemun-gu, Seoul 02447, Korea

**Keywords:** leelamine, malignancies, molecular mechanisms, preclinical models

## Abstract

For the last couple of decades, natural products, either applied singly or in conjunction with other cancer therapies including chemotherapy and radiotherapy, have allowed us to combat different types of human cancers through the inhibition of their initiation and progression. The principal sources of these useful compounds are isolated from plants that were described in traditional medicines for their curative potential. Leelamine, derived from the bark of pine trees, was previously reported as having a weak agonistic effect on cannabinoid receptors and limited inhibitory effects on pyruvate dehydrogenase kinases (PDKs). It has been reported to possess a strong lysosomotropic property; this feature enables its assembly inside the acidic compartments within a cell, such as lysosomes, which may eventually hinder endocytosis. In this review, we briefly highlight the varied antineoplastic actions of leelamine that have found implications in pharmacological research, and the numerous intracellular targets affected by this agent that can effectively negate the oncogenic process.

## 1. Introduction

Cancer remains a major cause of mortality on a global scale. Moreover, among the patients who respond positively to the treatment, most develop recurrence after a certain delay, leading to severe complications during therapy. Various bioactive natural products can exert significant beneficial effects in cancer treatment and prevention [1,2,3,4,5,6,7,8,9,10,11,12,13,14,15,16]. Indeed, natural products constitute an important source for the discovery of anticancer compounds, as they can modulate various important hallmarks of tumor cells, and several antineoplastic drugs approved by the United States’ Food and Drug Administration (FDA) over the last four decades are based primarily on natural products and/or their derivatives [4,7,8,13,14,17,18,19,20,21,22,23,24,25,26].

The process of lysosomotropism can be defined as the accumulation of compounds with basic and lipophilic properties inside the lysosomes. Lysosomes are acidic single-membrane-enclosed organelles filled with peptidases including cathepsins that are necessary for the digestion of macromolecules, including diverse xenobiotics, via different degradation pathways such as endocytosis, phagocytosis, and so on. Lysosomes are responsible for cellular homoeostasis, and recent studies have also shown their implication in nutrient sensing and regulation of cellular metabolism and proliferation. Lysosomotropism can lead to the disturbance of homeostasis and disorder in the lysosomes and endosomes that impacts various lysosome-dependent cellular processes, including autophagy and cholesterol trafficking and thus growth and proliferation, among others [27,28]. Interestingly, numerous lysosomotropic compounds have shown therapeutic properties and are mainly used in psychiatry as antidepressants and antipsychotics (e.g., chloropromazine, approved in 1999 by the FDA [29], and imipramine and Tofranil®, approved in 1959 by the FDA). Imipramine is a tricyclic compound derived from dibenzazepine; this antidepressant enables the inhibition of two neurotransmitters’ reuptake (serotonin and norepinephrine) within the synaptic cleft of the central nervous system (CNS) [30]. Moreover, imipramine is suggested not only for depression therapy, but can also be employed for childhood nighttime incontinence, a condition that can cause psychological distress in children [30]. Interestingly, lysosomotropic products may be used as antimalarial compounds (chloroquine phosphate, ARALEN®, approved in 1949 by the FDA). Interestingly, chloroquine was applied to treat malaria and other inflammation-associated disorders initially [31], but the discovery of the autophagy process led to its potential application in cancer.

Thereafter, significant lysosomal damage was highlighted in animals receiving chloroquine, and thus it was classified as a lysosomotropic. It could also readily penetrate the lysosomal membrane, thus causing its accumulation within the lysosome [32]. Other lysosomotropic agents such as estrogen receptor antagonists (tamoxifen and Nolvadex®, approved in 2002 by the FDA), may exhibit both estrogenic agonistic and antagonistic effects in different parts of the body. Due to these antagonistic properties, it can be used as a selective estrogen receptor modulator. In the breast tissue, it acts as an estrogen antagonist and causes antiestrogenic and anti-breast cancer effects. Through downstream intracellular processes, it can modulate processes occurring during the cell cycle, which also leads to its classification as a cytostatic compound. In the bone, tamoxifen can stimulate estrogen receptors instead of blocking them, thus exerting an estrogen agonistic effect, and may prevent osteoporosis in postmenopausal women [33,34].

Interestingly, recent studies have also demonstrated that tumor cells were sensitive to some lysosomotropic products, and this property has been described as a promising approach for the selective inhibition of tumor cells. Among such compounds, siramesine has shown notable anticancer effects with nonsignificant toxicity targeting diverse tumor cell lines [35]. Siramesine, at concentrations above 20 µM, has been shown to induce a loss of matrix metallopeptidases (MMPs), apparently by a direct destabilization of the mitochondrial membrane, and afterwards by triggering cell death, both apoptotic and nonapoptotic. Interestingly, at concentrations below 20 µM, a slower cell death occurred, linked to the gradual deterioration of cellular homeostasis due to interference in diverse activities, such as lysosomal degradation, autophagic flux, intracellular trafficking, and energy metabolism [35]. 

Topotecan, a topoisomerase inhibitor can be applied for the treatment of metastatic ovarian cancer as well as that of platinum-sensitive relapsed small cell lung cancer [36]. This agent was also recently approved for therapy of stage IVB recurrent or persistent cervical cancer [37]. Topotecan can attach to topoisomerase I and form a complex with DNA, thus causing breaks during DNA replication and therby promote cell death [38]. Moreover, topotecan has also exhibited symptom palliation in small cell lung cancer (SCLC) patients, leading to an improvement in dyspnea, anorexia, hoarseness, fatigue, and interference with daily activities [39,40]. Sunitinib malate is a multitargeted tyrosine kinase inhibitor that can display significant anticancer effects. It has also been tested for its possible lysosomotropic property that may also confer significant anticancer activity, and has shown promising results in human cervical cancer cells [41,42].

## 2. Chemistry

**Figure 1:**
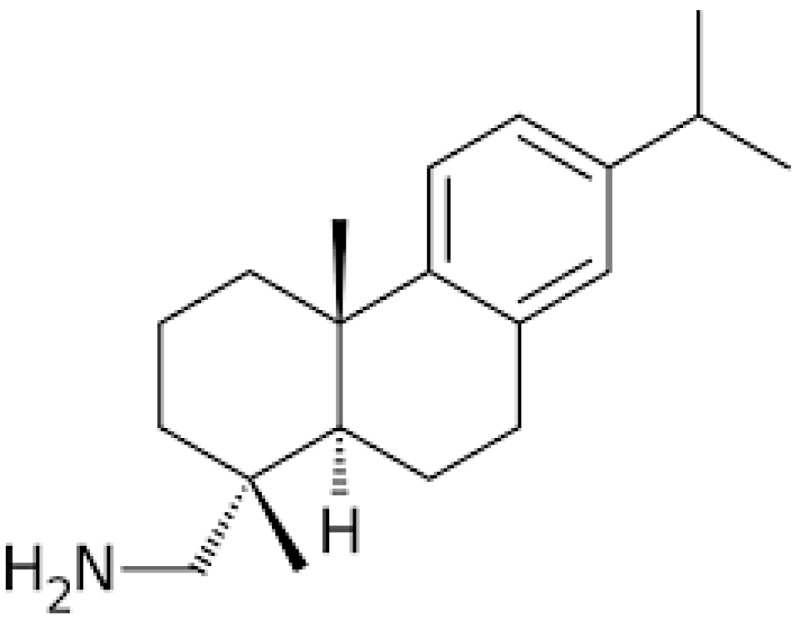
The chemical structure of leelamine.

Among the identified lysosomotropic compounds, leelamine (dehydroabietylamine), a lipophilic diterpene amine phytochemical with a pKa of 9.9, is a natural compound extracted from the bark of pine trees [28,43]. Leelamine (structure shown in Figure 1), which was previously reported as having a weak agonistic effect on cannabinoid receptors and limited inhibitory effects on pyruvate dehydrogenase kinases (PDKs) [28,44], has caught particular attention for its lysosomotropic property and significant inhibitory effects on proliferation and tumorigenesis [43,45,46] in human breast cancer cells [47], melanoma cell lines [28,43,48,49], and prostate cancer cell lines [50]. A recent study, during which chemical modifications of leelamine were made to identify its active site responsible for its anticancer activities, has shown that a suppression of the amino group or its charge may lead to a deletion of its antineoplastic actions, thus indicating that its lysosomotropic as well as its antitumorigenic effect may be mediated by this moiety [28]. Furthermore, a recent discovery made by Sehrawat et al. has also suggested a novel potential application of leelamine as a potent antidiabetic agent through the induction of cytochrome P450 2B6 (CYP2B) activity [51].

## 3. Anticancer Effects of Leelamine

Malignant melanoma has the ability to undergo rapid metastasis and also an aptitude for developing chemotherapeutic resistance [52]. V600E-BRAF is a mutant of the B Rapidly Accelerated Fibrosarcoma protein with a aline residue replaced by glutamic acid in the kinase domain, which leads to an upregulation of the kinase activity and drug resistance development. Among the used treatments, zelboraf, tafinlar, and mekinist can target the deregulated MAPK pathway [53,54]. Nevertheless, those therapeutic strategies have seen their efficiency limited by drug resistance that causes the evolution of further aggressive melanomas [55,56].

In melanoma cancer cell lines treated with leelamine, an inhibition of tumor development without significant systemic toxicity has been shown using various techniques, including modulation transfer spectroscopy MTS [3-(4,5-dimethylthiazol-2-yl)-5-(3-carboxymethoxyphenyl)-2-(4-sulfophenyl)-2H-tetrazolium] assay and liquid chromatography–mass spectrometry [43]; furthermore, a decrease in the proliferation and vascular development has been highlighted [48]. Nanolipolee-007, a liposomal form of leelamine, was also shown to reduce melanoma metastasis and to induce apoptosis [49]. Prostate cancer represents 25% of all cancers diagnosed in men and its incidence continues to rise in many countries; it is also the fifth most frequent cause of mortality among all the cancer types in men [57,58,59,60]. Castration-resistant prostate cancer (CRPC) resulting from androgen withdrawal has been found to be quite challenging to cure due to its resistance to testosterone-suppression therapy, which is supposed to be the most efficient castration treatment [61].

Indeed, CRPC can be driven by aberrant activation of the androgen receptor (AR) by mechanisms varying from its amplification, mutation, and post-translational modification to expression of splice variants, including AR-V7. Leelamine treatment of prostate cancer cell lines LNCaP (an androgen-responsive cell line), C4-2B (an androgen-insensitive variant of LNCaP), and 22Rv1 (a CRPC cell line that express AR-Vs), and a murine prostate cancer cell line, Myc-CaP, led to decreased mitotic activity and prostate-specific antigen expression, in addition to apoptosis induction that caused cell death [50]. Breast cancer is major first cause of cancer mortality among women at a global scale, and even though the effectiveness of the existing multidisciplinary approaches to treat breast cancer has been proved, its therapy still remains a great medical challenge [18,19,20,21]. In MDA-MB-231, MCF-7, and SUM159 breast cancer cells, it has been shown that leelamine provoked cancer cell death through an apoptotic process in a dose-dependent fashion, while a normal mammary epithelial cell line (MCF-10A) was found to be completely resistant to the apoptotic and cell growth-inhibiting effects of the drug. Interestingly, it has been demonstrated that leelamine was also able to suppress breast cancer stem cells’ self-renewal ability substantially [47].

## 4. Molecular Targets Affected by Leelamine

The accumulation of leelamine in late endosomes or lysosomes provokes the blocking of cholesterol translocation from the lysosomes to cytoplasm, due to a lack of unbound cholesterol, making the latter unavailable for the cancer cells’ activities. Thus, leelamine indirectly targets various cellular and oncogenic signaling pathways [28,48] (Table 1). In fact, free cholesterol is a main factor for numerous cellular functions, including receptor-mediated endocytosis. In the cell, cholesterol concentrations are maintained in balance by the transfer of bound forms of low-density lipoprotein (LDL) from the cell membrane to lysosomes, where it can be hydrolyzed to free cholesterol, followed by cholesterol transport to the cytosol by Niemann–Pick type C proteins (NPC) [28,43,49]. When trapped in the lysosome, and thus not available for cellular processes, autophagic flux and receptor-mediated endocytosis are inhibited [62,63], which is also associated with the inhibition of receptor tyrosine kinases RTK signaling pathways, which can suppress the activation of the downstream PI3K/AKT, STAT3, and MAPK signaling cascades [4,28] that are responsible for cancer progression. Indeed, previous findings have shown that cellular growth is modulated mainly by different factors including diverse tumor suppressors and proto-oncogenes/oncogenes, as well as signaling molecules such as PI3K and Akt [64].

PI3K is a lipid kinase that is activated by receptor tyrosine kinases, resulting in the expression of a crucial secondary messenger, phosphatidylinositol-3,4,5-trisphosphate, and consequently enabling protein kinase B PKB to be activated, which can function as a prosurvival molecule [65,66,67,68]. Also, to generate membrane type 1 matrix metalloproteinase (MT1-MMP), which can regulate extracellular matrix remodeling [24,69], an important role is played by the PI3K–Akt pathway [70]. MT1-MMP can further lead to the overexpression of vascular endothelial growth factor expression and thus mediate angiogenesis [71]. The interaction of extracellular matrix with cells has a key role in cancer metastasis [72]. Among the ubiquitous cytoplasmic tyrosine kinases, focal adhesion kinase (FAK) represents a key modulator of signaling by integrins, which are the main cellular receptors responsible for interacting with various extracellular proteins [73,74].

MMP-9, which has been implicated in the degradation of the extracellular matrix, has an important function in cancer invasion and metastasis, and its expression is modulated by numerous growth factors, including TGF-β1 in various cell types [75]. FAK can also be expressed as a consequence of the alteration of an autoinhibitory intramolecular interaction between its amino terminal FERM (protein 4.1R, ezrin, radixin, moesin) domain and the central kinase domain. The activation of FAK leads to the formation of a complex with Src family kinases, which can initiate several downstream signaling pathways that can regulate various tumorigenic processes including metastasis [73]. Interestingly, FAK was shown to be overexpressed in diverse tumor cell lines such as human colorectal cancer [76,77].

The MAPK/ERK pathway is one of the major cascades functioning downstream of the FAK pathway. Once a ligand has been bound to the membrane RTK, a signal is transmitted, leading to the translocation of ERK (MAPK) to the nucleus, where it may activate transcription factors that control gene expression [20,56]. Signal transducers and activators of transcription (STATs) are prominent proteins involved in a variety of crucial cellular functions associated with proliferation, survival, and angiogenesis. Within different STAT members, STAT3 is regularly overexpressed in cancer cells and can modulate the expression of various oncogenic genes controlling the growth and metastasis of malignant cells [12,59,60,78,79,80,81,82,83,84,85]. This protein can be persistently activated in diverse tumor cells or may be induced upon exposure to cytokines, growth factors, and other stimuli [78,79,80,81,82,83,84,85] and can drive the tumorigenic process. The detailed effects of leelamine against several major cancers are briefly discussed below.

### 4.1. Melanoma

In the metastatic melanoma cell line UACC 903, the first study carried out by Kuzu et al. indicated that leelamine oil dissolved in dimethyl sulfoxide (DMSO) caused cholesterol accumulation and modified subcellular cholesterol localization, combined with an alteration in the members of the RTK–AKT/STAT3/MAPK signaling cascades (Erk, CREB, and RPS6KB1 (p70S6K), as well as activation of the STAT3 pathway, and phosphorylation of EIF4EBP1 (4E-BP1) was attenuated post-treatment), and the Akt/mTOR cascade was also inhibited. Another study led by Gowda et al. highlighted that leelamine decreased the proliferation and vascular development of melanoma cancer cells and increased apoptosis by initiating programmed cell death mediated through a G0–G1 block and causing fewer cells to assemble in the S-phase of the cell cycle. Those observations were induced by the inhibition of the PI3K/Akt, MAPK, and STAT3 pathways through the suppression of intracellular cholesterol transport, and similar effects were noted in preclinical models. Interestingly, negligible toxicity has been observed both in vitro and in vivo. Furthermore, no modifications in cellular morphology of vital organs have been observed after 3 weeks of treatment [48]. Recently, a group of scientists developed a nanoparticle which is a liposomal form of leelamine, called Nanolipolee-007 that was shown to reduce melanoma metastasis and to induce apoptosis independently from the BRAF proto-oncogene mutational status of the circulating tumor cells. Interestingly, leelamine exposure also led to cholesterol accumulation in lysosomes, which can abrogate receptor-mediated endocytosis, endosome trafficking, and affect the AKT pathway, which is decisive in circulating tumor cell survival [49].

### 4.2. Prostate Cancer

Leelamine-treated human prostate cancer cell lines exhibited decreased mitotic activity and prostate-specific antigen expression, in addition to apoptosis induction that led to cancer cell death. These results were combined with the inhibition of mRNA and protein expression levels of AR as well as its splice variants, including AR-V7. In silico studies suggest an interaction of leelamine with Y739 in AR that would be responsible for the loss of function. In a 22Rv1 xenograft treated by leelamine, growth inhibition was observed, caused by a significant decrease in Ki-67 expression, mitotic activity, AR variant expression, and secretion of prostate-specific antigen PSA [50].

### 4.3. Breast Cancer

**Figure 2:**
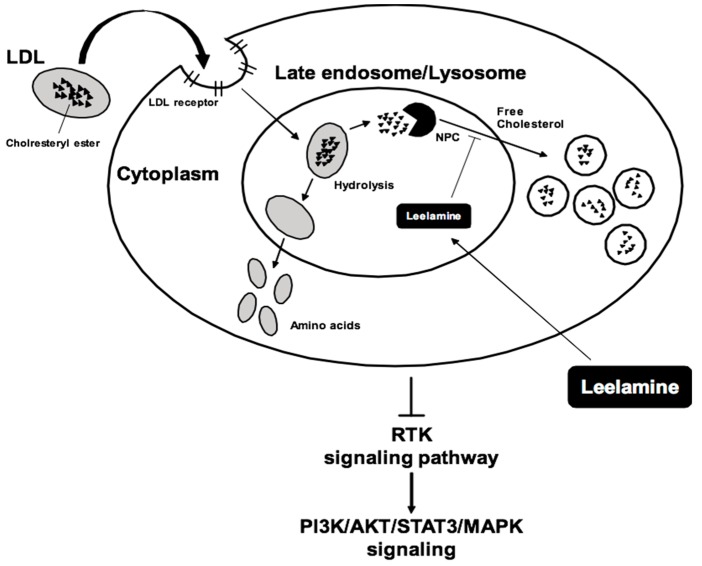
The potential mechanism(s) of action of leelamine. LDL: low-density lipoprotein; NPC: Nuclear Pore Complex.

Leelamine has been reported to mitigate growth and induce programmed cell death in a variety of breast cancer cell lines. These observations were combined with the induction and/or activation of the multidomain proapoptotic proteins Bax and Bak, caspase-9 activation, and cytosolic release of cytochrome c. Interestingly, in orthotopic SUM159 xenografts in mice, leelamine suppressed tumor growth without displaying any systemic toxicity [47]. The diverse targets affected by leelamine have been summarized in Figure 2.

## 5. Other Important Pharmacological Actions

Cytochrome P450 (CYP) is a key enzyme, which is considered as the most plentiful phase I drug-metabolizing enzyme in humans and can catalyze xenobiotic compounds such as toxic products or medicines. Recent studies have shown that diabetes is related with a notable reduction in hepatic P450 3A4 enzymatic activity and protein level [51,86]. In leelamine-treated male mice liver, the activity of CYP2B increased almost 4-fold compared to control groups. Furthermore, leelamine significantly increased CYP2B10 protein levels in liver. Interestingly, no major changes in the CYP2B mRNA levels post-treatment were observed, thereby suggesting that leelamine is a novel inducer of CYP2B activity in vivo, which may open up new perspectives for its development for diabetes treatment [51].

## 6. Metabolism and Toxicity Studies

It has been discovered that both in vitro and in vivo, leelamine undergoes only phase 1 metabolism, producing a mono-hydroxylated metabolite at the C9 carbon of the octahydrophenanthrene ring (M1). CYP 2D6 was identified to be the dominant CYP enzyme that allows the biotransformation of leelamine to its hydroxylated metabolite, in comparison to the other major isoforms. Interestingly, only one metabolite has been identified so far in urine, and none in feces, thus suggesting that leelamine is metabolized to a mono-hydroxyl metabolite by CYP2D6 and principally excreted in the urine [87]. 

Body weights of mice inoculated with melanoma tumor cells following leelamine treatment demonstrated no significant differences compared to the control groups, even after daily treatment for 22 days. Also, after this period, blood parameters were measured and no organ-related toxicity was noticed. Moreover, after histological analysis, no modifications in the morphology or overall structure of the liver, spleen, kidney, intestine, lung, or heart was observed [43,48]. Interestingly, intraperitoneal administration of leelamine in mice (7.5 mg/kg; 5 times/week) led to the suppression of the orthotopic SUM159 xenografts’ growth without any significant toxicity [45].

## 7. Conclusions

This review summarizes in brief the limited reports related to the antineoplastic effects of leelamine. The prior studies have reported about the potential antitumor efficacy of leelamine, resulting from its lysosomotropic property, as well as its impact on tumor development (mitigation of tumor cell proliferation, metastasis, and induction of apoptosis and/or autophagy). Furthermore, leelamine has been shown to target key oncogenic pathways (RTK–AKT/STAT3/MAPK, AKT/mTOR) in various tumor cells. Taken together, these results make a case for further investigations to elucidate the exact mechanisms underlying the intracellular disruption of cholesterol homeostasis induced by leelamine. Additionally, more information related to the chemistry of its amino group that has been shown to be responsible for its proautophagic and thus anticancer properties needs to be determined. This may open new perspectives for the development of lysosomotropic compounds, especially leelamine, as novel anticancer drugs.

## Figures and Tables

**Table 1:** Reported pharmacological effects of leelamine.

Diseases	In Vitro/ In Vivo	Types	Pathways/ Molecules Altered	Concentration Range Tested	IC_50_	References
**Anticancer Effects**	**In Vitro**	Melanoma (UACC 903; 1205 Lu)	RTK–AKT/STAT3/MAPK ↓, Erk ↓, CREB, RPS6KB1 p70S6K ↓ and STATs↓, phosphorylation of EIF4EBP1 (4E-BP1) ↓, mTOR ↓ G0–G1 ↓ PI3K ↓ NPC1 ↓	0 µM, 6–100 µM	UACC 903: 1.35 ± 0.1 1205 Lu: 1.93 ± 0.2	[28,43,48]
		Breast cancer (MDA-MB-231, MCF-7; SUM159)	Bax and Bak ↑, caspase-9 ↑, cytochrome *c* ↑	0–5 µM	N.D.	[47]
		Prostate cancer (LNCaP; C4-2B; 22Rv1)	AR ↓	N.D.	N.D.	[50]
	**In Vivo**	Nude mice expressing UACC 903; 1205 Lu melanoma cells	tumor size ↓ by 50% proliferation ↓	80 mg/kg body weight	N.D.	[43]
		Female nude mice (SUM159 xenograft breast cancer)	tumor size ↓ by 70%	7.5 mg/kg body weight	N.D.	[47]
		22Rv1 xenograft (prostate cancer)	tumor growth ↓ PSA secretion ↓	N.D.	N.D.	[50]
**Antidiabetic Effects**	**In Vivo**	male mice liver	CYP2B increased ↑ CYP2B10 ↑	5, 10, or 20 mg/kg	N.D.	[51]

Abbreviations: Akt: Phosphorylated Protein kinase B. Bcl-2: B-cell lymphoma 2. Bax: Bcl-2-associated X protein. c-Myc: proto-oncogene. STAT3: Signal transducer and activator of transcription 3. ↑: Upregulation. ↓: Downregulation. RTK: Receptor tyrosine kinase. MAPK: Mitogen-activated protein kinases. ERK: Extracellular signal-regulated kinases. CREB: C-AMP response element-binding protein. RPS6KB1: Ribosomal Protein S6 Kinase B1. p70S6K: Ribosomal protein S6 kinase beta-1. EIF4EBP1: Eukaryotic Translation Initiation Factor 4E Binding Protein 1. EIF4E: Eukaryotic translation initiation factor 4E. MTOR: Mechanistic target of rapamycin. PI3K: Phosphatidylinositol 3-kinases. NPC: Intracellular Cholesterol Transporter 1. BAX: Bcl-2-associated X protein. BAK: BCL2 antagonist/killer. AR: Androgen Receptor. CYP2B: Cytochrome P450 2B6.

## Abbreviations

pAkt	Phosphorylated protein kinase B
AR	Androgen-responsive
Bax	Bcl-2-associated X protein
CREB	C-AMP response element-binding protein
CYP2B10	Cytochrome P450, family 2, subfamily b, polypeptide 10
CYP2B	Cytochrome P450 2B6
EIF4EBP1	Eukaryotic translation initiation factor 4E-binding protein 1
ERK	Extracellular signal-regulated kinases
FAK	Focal adhesion kinase
FERM	Protein 4.1R, ezrin, radixin, moesin
Hsp90	Chaperone
mTOR	Mechanistic target of rapamycin
LDL	Low-density lipoprotein
MT1-MMP	Membrane type 1 metalloproteinase
MAPK	Mitogen-activated protein kinases
NPC	Niemann–Pick type C
PI3K	Phosphatidylinositol 3-kinases
p70S6K	Ribosomal protein S6 kinase beta-1
PDK	Pyruvate dehydrogenase (acetyl-transferring) kinase
RTK	Receptor tyrosine kinase
RPS6KB1	Ribosomal protein S6 kinase B1
TGF-β1	Transforming growth factor
STAT3	Signal transducer and activator of transcription 3
